# Relations among questionnaire and experience sampling measures of inner speech: a smartphone app study

**DOI:** 10.3389/fpsyg.2015.00517

**Published:** 2015-04-27

**Authors:** Ben Alderson-Day, Charles Fernyhough

**Affiliations:** Department of Psychology, Durham UniversityDurham, UK

**Keywords:** covert speech, dialog, introspection, verbal thinking, self-talk

## Abstract

Inner speech is often reported to be a common and central part of inner experience, but its true prevalence is unclear. Many questionnaire-based measures appear to lack convergent validity and it has been claimed that they overestimate inner speech in comparison to experience sampling methods (which involve collecting data at random timepoints). The present study compared self-reporting of inner speech collected via a general questionnaire and experience sampling, using data from a custom-made smartphone app (*Inner Life*). Fifty-one university students completed a generalized self-report measure of inner speech (the Varieties of Inner Speech Questionnaire, VISQ) and responded to at least seven random alerts to report on incidences of inner speech over a 2-week period. Correlations and pairwise comparisons were used to compare generalized endorsements and randomly sampled scores for each VISQ subscale. Significant correlations were observed between general and randomly sampled measures for only two of the four VISQ subscales, and endorsements of inner speech with evaluative or motivational characteristics did not correlate at all across different measures. Endorsement of inner speech items was significantly lower for random sampling compared to generalized self-report, for all VISQ subscales. Exploratory analysis indicated that specific inner speech characteristics were also related to anxiety and future-oriented thinking.

## Introduction

“*Human beings talk to themselves every moment of the waking day*” (Baars, 2003, p. 106)

Inner speech—talking to oneself silently and internally—seems to be a central part of conscious experience. Cognitive and developmental research on inner speech has led to it being associated with a variety of activities and skills, including problem-solving, memory, and self-reflection (Sokolov, 1975; Morin, 2005; Perrone-Bertolotti et al., 2014). Less information exists, however, on the extent and nature of everyday inner speech use, in part because of methodological difficulties in measuring the phenomenon reliably.

Studies seeking to empirically investigate inner speech have tended to use generalized self-report methods, such as “thought-listing,” diaries, or questionnaires. For example, Morin et al. (2011) asked a sample of 380 university students to list, in an open format, “as many verbalizations as they typically address to themselves.” They then coded responses for their content (such as positive or negative statements, or comments about other people) and their apparent function (such as self-regulation). By far the most common kind of content reported was self-talk about oneself, including statements relating to self-evaluation and utterances concerning emotions, personal relationships, and physical appearance. In terms of functions, inner speech was most commonly used for planning ahead, remembering previous events, and motivating behavior. These findings were consistent with a diary-based study of future-thinking by D’Argembeau et al. (2011): participants were asked to list a selection of thoughts each day, and rate their content and phenomenological qualities, including whether they were in inner speech or in other modalities (such as visual imagery). Compared to other modalities, inner speech was particularly associated with planning and decision-making. In addition, inner speech was more likely to occur for negative or neutral thoughts than positive thoughts.

Broadly similar results have been provided by questionnaire-based studies of inner speech and private speech (i.e., speech which is external but self-directed). Duncan and Cheyne (1999) developed a questionnaire to study self-verbalizations (a mixture of private and inner speech) and examined the most common factors that arose in students. Overall, verbalizations were most often used for “cognitive-attentional” activities (i.e., trying to remember something, or avoid distractions) and organizing behavior, such as planning out a series of actions. Brinthaupt et al.’s (2009) Self-Talk Scale (STS) is a measure that emphasizes inner speech more specifically, and includes four main factors: social assessment, self-reinforcement, self-criticism, and self-management. Across a series of experiments, they showed that individual differences in self-talk frequency related to various cognitive, behavioral, and mood factors. For instance, those who reported more frequent instances of critical or evaluative self-talk also reported lower self-esteem and a greater number of automatic negative thoughts.

Most recently, McCarthy-Jones and Fernyhough (2011) devised the Varieties of Inner Speech Questionnaire (VISQ). The VISQ asks about variations in inner speech that reflect its putative origin in external communication (Vygotsky, 1987), whereby silent or covert self-talk reflects an internalized and transformed version of outer dialog and social interaction. Thus, it asks about inner speech that varies in structure (such as being in full sentences, or single words, or having a turn-taking quality), identity (such as involving interaction with other people), and, consistent with other scales, self-regulatory behaviors (such as encouraging or criticizing oneself). McCarthy-Jones and Fernyhough’s (2011) initial use of the scale displayed a four-factor structure, encompassing *dialogic* inner speech (inner speech with a conversational quality), *evaluative/motivational* inner speech (i.e., self-regulatory inner speech), *other people* in inner speech (e.g., comments from other agents, such as relatives), and *condensed* inner speech (the extent to which inner speech was abbreviated in some way). They found that dialog-like and evaluative characteristics of inner speech were very common (endorsed by 75–80% of participants), while the presence of other people in inner speech and condensed inner speech also appeared in a substantial minority of individuals. Moreover, the different characteristics of inner speech were specifically related to differing aspects of psychopathology: for instance, McCarthy-Jones and Fernyhough (2011) observed that inner speech containing other people or evaluative characteristics was related to self-reported scores for anxiety, while a separate study by Alderson-Day et al. (2014) found that evaluative inner speech was also associated with lower self-esteem. Taken together, the above studies point to an experience of everyday inner speech that often involves self-evaluation and thinking about the future, but can sometimes be associated with low mood and feelings of anxiety.

However, the validity of investigating inner speech in this way—that is, relying on participants’ generalized self-reports—has been questioned. Using the same sample as Morin et al. (2011), Uttl et al. (2011) assessed the reliability and validity statistics of their own self-report method, along with Duncan and Cheyne’s (1999) Self-Verbalization Questionnaire, Brinthaupt et al.’s (2009) STS, and two other self-talk measures: the Inner Speech Scale (ISS; Siegrist, 1995) and the Self-Talk Inventory (Calvete et al., 2005). While all of the scales showed good internal reliability, they showed very little convergent validity (with only the STS and ISS being more than weakly correlated) and tended not to correlate at all with the inner speech reports collected by Morin et al. (2011). That is, what people freely list as generally occurring in their inner speech, and how they respond to various general questionnaires about the same topic, did not seem to closely correspond.

A second, related cause for concern is the possibility that individuals may over-estimate their own inner speech when they are asked about it in generalized terms. While some older studies have claimed very high frequency rates for inner speech, i.e., >50% of daily samples (Klinger and Cox, 1987; Goldstein and Kenen, 1988), it has recently been suggested that inner speech is far from ubiquitous and universal. Based on their studies with Descriptive Experience Sampling—a method where participants are prompted at random by a beeper to record their everyday inner experience, and are then interviewed about that experience in depth—Heavey and Hurlburt (2008) argued that inner speech only occurs in 26% of random samples. Hurlburt et al. (2013) suggest that inner speech questionnaires over-estimate the occurrence of inner speech because they ask participants to provide a general estimate of its occurrence, which is more likely to reflect participants’ preconceptions about their inner speech rather than its actual presence on a moment-by-moment basis. Hurlburt et al.’s (2013) view is supported by evidence from ecological momentary assessment studies, which have often reported over-estimation of traits and behaviors when they are gathered via generalized self-report compared to random or momentary sampling (see Shiffman et al., 2008, for a review).

As has been noted elsewhere (McCarthy-Jones and Fernyhough, 2011; Morin et al., 2011), new and alternative methods are needed to fully assess whether generalized self-report measures of inner speech can be reliable and valid indicators of everyday inner speech (Alderson-Day and Fernyhough, 2014). One way to do that is to combine questionnaire methods with experience sampling (Csikszentmihalyi and Larson, 1987), in which participants are prompted at random intervals to provide data. DES as used by Hurlburt et al. (2013) is one kind of in-depth experience sampling, but it is very resource-intensive and typically only used in small groups of participants. An alternative option is to use smartphone-based assessments to gather large amounts of randomly sampled data. Experience sampling via iPhones and other devices has previously been used successfully to examine the relations between mood and mind-wandering (Killingsworth and Gilbert, 2010). In some cases, such methods have also been reported to change participants’ self-reporting skills (a phenomenon known as ‘reactivity’): for instance, Runyan et al. (2013) observed increased levels of self-awareness and better time management in a group of university students who were assigned to use an experience sampling app (*iHabit*) compared to controls who simply completed general questionnaires.

We applied the smartphone app methodology to the study of inner speech using a custom-made app, *Inner Life*. Inner Life works by prompting participants at random, twice a day, to answer a short series of questions about their ongoing thoughts, feelings, and behavior. Specifically, volunteers are asked to indicate what they were doing immediately prior to noticing the alert from the app. In the present study they did this for 2 weeks, building up a maximum of 28 data points for various aspects of inner life, including inner speech, activity, mood, and mind-wandering.

In addition, we asked participants to complete a generalized measure of inner speech—the VISQ (McCarthy-Jones and Fernyhough, 2011)—at the start and end of the two-week period. The VISQ was chosen for the following reasons: (1) it is unique among questionnaire measures in asking specifically about inner speech, rather than more general “self-talk” (which could include overt and covert verbalizations); (2) it covers a broad range of phenomenological features of inner speech, with relevance to form, function, and identity; and (3) its subscales show good internal reliability and reasonable validity, in terms of correlations with other self-report traits (such as proneness to anxiety).

We hypothesized that (1) generalized endorsements of inner speech characteristics would be reliable indicators of inner speech incidence recorded via random sampling, but that (2) moment-by-moment endorsements of inner speech would, on average, be lower than generalized endorsements of inner speech, based on the claims of Hurlburt et al. (2013).

We also set out to explore how inner speech related to other measures of mood and thinking identified in previous studies on the topic. First, based on prior links between inner speech, self-reflection, anxiety, and positive and negative thinking (D’Argembeau et al., 2011; McCarthy-Jones and Fernyhough, 2011; Morin et al., 2011; Alderson-Day et al., 2014), we included mean scores for happiness and anxiety in this analysis. Second, we assessed relations between inner speech and temporal thinking (i.e., whether or not participants were generally thinking about the past, present, or future) based on the apparent involvement of inner speech in remembering the past and planning/future thinking (D’Argembeau et al., 2011). If inner speech served the apparent temporality-related functions reported by D’Argembeau et al. (2011) and Morin et al. (2011), then different characteristics of the VISQ could be expected to be associated with temporal thinking about the past and future.

## Materials and Methods

### Participants

Fifty-one university students (38 female; age *M* = 19.88, SD = 2.96) were recruited via a participant-pool advertisement. All participants had English as their first language. Participation was rewarded with course credit. All procedures were approved by a local university ethics committee.

### Procedure

Participants were provided with a link to download Inner Life via an online information and consent page. Inner Life was made available to participants for Android phones (via a private link) or iOS (via the *Testflight* app-testing service). The app download page included instructions on how to use the app and respond to each alert. Participants were encouraged to respond to the app as soon as it was safe to do so, and to answer based on what was happening immediately prior to the moment they noticed the alert.

**FIGURE 1:**
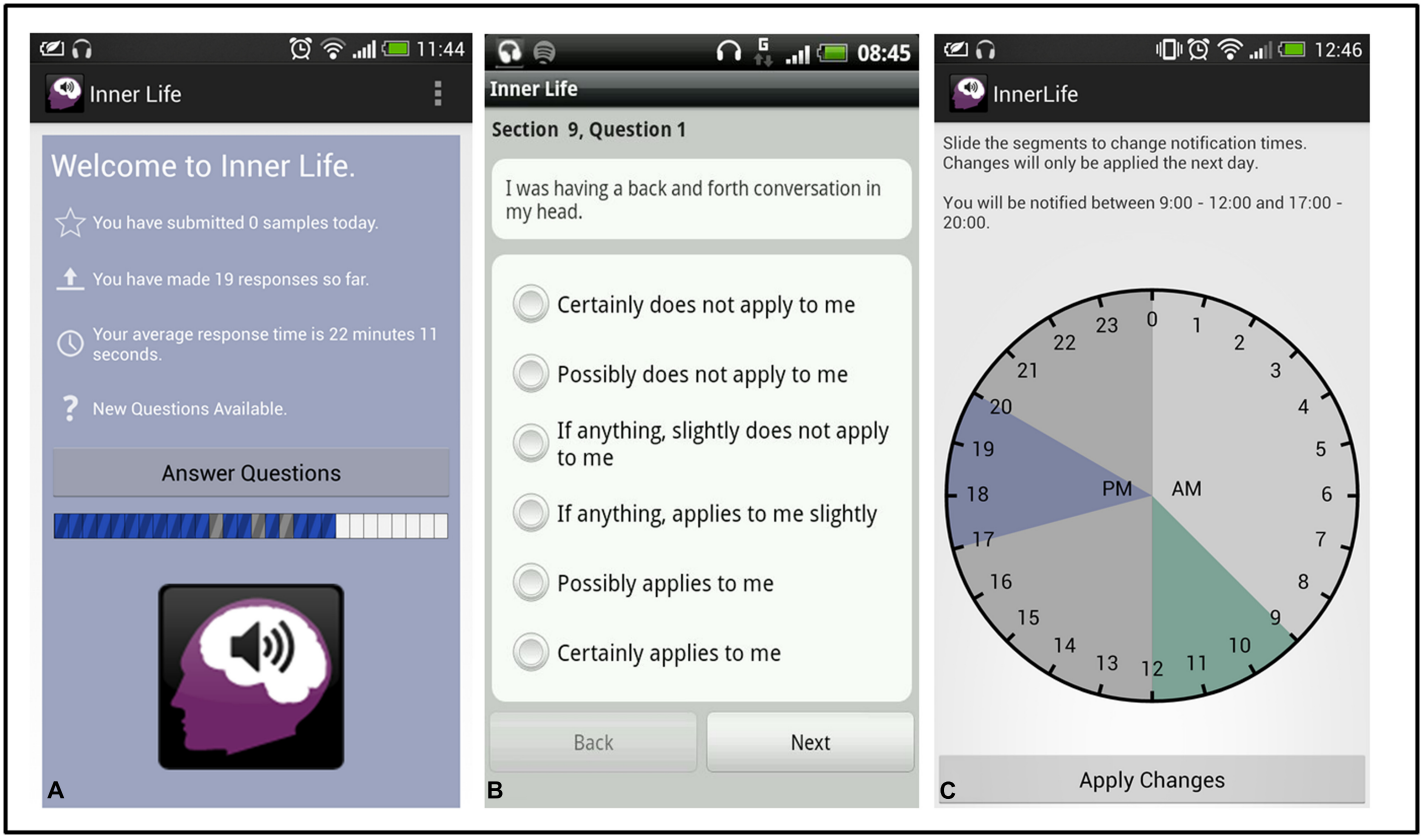
**Inner Life.** Participants received two alerts a day **(A)** to answer a short battery of questions on inner speech and related topics (B). Alerts would occur at random within two 3-hour windows, selected by the participant **(C)**.

When the app was first opened, participants were prompted to complete a battery of generalized questionnaire measures, taking roughly 5–10 min to complete (including the full VISQ). Following this, Inner Life was configured to deliver two alerts a day for 14 days (see **Figure 1**). Each alert contained 12–18 questions about ongoing mental phenomena, taking less than 2 min to complete. The alerts occurred at random intervals within two 3-h windows each day, one early and one late (by default, this was set to 9 am–12 pm and 2 pm–5 pm). Participants could choose when their windows occurred to avoid intrusion. The only limit on window selection was that one had to be before 2 pm, and one after (in order to ensure a spread of responses between morning and afternoon). On the 14th day of testing, the final alert contained a second general self-report battery for assessment of test–retest reliability.

### Measures

Inner Life collected a large battery of mood and psychopathology data that are being analyzed as part of a larger ongoing study. Here, we report the main variables associated with inner speech.

#### Inner Speech via General Questionnaire

The VISQ (McCarthy-Jones and Fernyhough, 2011) was used to assess phenomenological characteristics of inner speech at the start and end of the two-week period. The VISQ is an 18-item scale containing four subscales: *Dialogic Inner Speech* (Dialogic henceforth), *Evaluative/Motivational Inner Speech* (Evaluative), *Other People in Inner Speech* (Other People), and *Condensed Inner Speech* (Condensed). Each item is answered on a 6-point Likert scale ranging from “Certainly does not apply to me” (1) to “Certainly applies to me” (6). Each of the subscales has very good internal reliability (Cronbach’s alpha > 0.80) and moderate/good test–retest reliability (>0.6). Participants completed the full VISQ on entry to the study (T1) and at the end of the study (T2).

#### Inner Speech via Random Sampling

Inner speech collected via the random alerts was assessed using four adapted items from the full VISQ.

For each subscale the highest loading item from the original VISQ factor analysis by McCarthy-Jones and Fernyhough (2011) was selected and then reworded to refer to the current moment:

At the time of the alert:
(1) *I was having a back and forth conversation in my head* (Dialogic)
(2) *My thinking was shortened compared to my normal, out-loud speech* (Condensed)
(3) *I was having the experience of other people’s voices* (Other People)
(4) *I was evaluating my behavior using my inner speech* (Evaluative)

Each item was presented sequentially, with participants answering using the same 6-point Likert scale as the full VISQ.

#### Other Measures

A selection of alerts also included ratings for three other relevant variables: happiness, anxiety, and temporal thinking.

• For **happiness** and **anxiety** participants indicated their current mood level on a visual analog scale, from 0–10. 50% of alerts contained happiness questions, while 75% contained anxiety questions. Questions about each were evenly spaced through the 14-day sampling period.
• For **temporal thinking**, participants were asked to indicate whether they were thinking about the past, present, or future at the moment of the alert. Fifty percentage of samples contained a question about temporal thinking. Samples with a temporal thinking question alternated each day (i.e., Day 1 contained a question in the AM window, Day 2 in the PM window).

### Analysis

As the large majority of outcome variables were non-normally distributed, non-parametric tests were used (Spearman’s Rho and Wilcoxon Signed Ranks Test). To compare generalized endorsements and randomly sampled incidences of inner speech, mean scores were calculated from the random alerts for each VISQ factor and then scaled up to provide “total” scores. For example, a mean score of 4 on the Dialogic item would receive a total score of 16, based on the fact that the general VISQ contains four items in the Dialogic subscale. Bivariate correlations were used to examine reliability of T1 scores. Wilcoxon tests were used to compare overall levels of endorsement for inner speech characteristics. T1 and T2 general VISQ scores were then assessed for test–retest reliability and compared for overall score, to assess changes in reporting following use of the app. To control for multiple comparisons for the four VISQ subscales, alpha was adjusted to *p* < 0.0125 (i.e., 0.05/4), while *p*-values between 0.0125 and 0.05 were treated as trends (see “Overall Characteristics of Inner Speech,” “Similarities and Differences in Inner Speech,” and “Changes in Inner Speech Following Random Sampling”). Finally, mean responses for happiness, anxiety, and temporal thinking were used to explore relations with generalized and randomly sampled inner speech. As this final analysis was exploratory, results were treated as significant at *p* < 0. 05 (see “Relations to Mood and Temporal Thinking”).

## Results

### Overall Characteristics of Inner Speech

All participants provided a full set of T1 data and responded to at least 25% of their app alerts (7/28 samples). The mean percentage of alerts responded to was 63.14% (SD = 17.10, Range = 29–93). The retest of general VISQ at T2 was also completed by 36 participants (see **Table 1** for mean scores on the VISQ).

**Table 1:** Endorsement of inner speech characteristics at start of the study (T1), during random sampling, and at the end of the study (T2).

	T1 (*n* = 51)		Random sampling (*n* = 51)			T2 (*n* = 36)	
	*M*	(SD, Range)	*M*	(SD, Range)	*Mean* SD	*M*	(SD, Range)
Dialogic (Max 24)	17.53	(5.24, 6–24)	12.94	(4.52, 4–20.62)	1.44	16.50	(6.10, 4–24)
Evaluative (Max 24)	19.45	(3.78, 8–24)	12.06	(4.28, 4.36–21.45)	1.30	18.69	(3.96, 8–24)
Other People (Max 30)	13.75	(6.89, 5–26)	10.48	(5.99, 5–25.59)	0.96	14.28	(7.00, 5–28)
Condensed (Max 30)	15.20	(6.66, 5–29)	12.73	(6.02, 5–28.75)	0.99	14.08	(6.75, 5–27)

(i) Randomly sampled VISQ ratings could be non-integers due to averaging across samples; (ii) Mean SD reflects average standard deviation of within-subjects samples.

### Similarities and Differences in Inner Speech

Spearman correlations between generalized endorsements of inner speech and random sampling incident reports were significant for Condensed (*r* = 0.69,* df* = 49, *p* < 0.001) and Other People (*r* = 0.46, *df* = 49, *p* < 0.001), but only approached significance for Dialogic, given the use of an adjusted alpha value (*r* = 0.30, *df* = 49, *p* = 0.031). There was no correlation between generalized and random scores for Evaluative (*r* = 0.03, *df* = 49, *p* = 0.851).

When the total scores for inner speech were compared across generalized endorsements and randomly sampled reports, each of the subscales were significantly lower when an experience sampling method was used. As **Table 1** shows, the greatest discrepancies were for Dialogic (Wilcoxon’s *Z* = -4.88, *df* = 50, *p* < 0.001) and Evaluative (*Z* = -5.88, *df* = 51, *p* < 0.001), followed by Condensed (*Z* = -3.19, *df* = 48, *p* < 0.001) and Other People (*Z* = -2.82, *df* = 48, *p* = 0.005).

When scores were compared between subscales, T1 reports of Evaluative inner speech were significantly higher than scores for Other People (*Z* = -5.03, *df* = 48, *p* < 0.001), Condensed (*Z* = -3.54,* df* = 46, *p* < 0.001), and, at trend level, Dialogic (*Z* = -2.29, *df* = 47, *p* = 0.022). This was not the case under random sampling, where the only pairwise comparison observed to reach significance was between Dialogic and Other People (12.94 vs. 10.48, respectively; *Z* = -2.52,* df* = 51, *p* = 0.012). In addition, the average discrepancy between T1 and random-sampling was greatest for Evaluative scores compared to all other subscales (all *p* < 0.002).

### Changes in Inner Speech Following Random Sampling

Test–retest reliability for T1 to T2 generalized endorsements of inner speech were significant and within an acceptable range for Dialogic (*r* = 0.81, *df* = 34, *p* < 0.001), Condensed (*r* = 0.88, *df* = 34,* p* < 0.001), and Other People (*r* = 0.68, *df* = 34, *p* < 0.001). Again, Evaluative was much less reliable, showing only a modest correlation between T1 and T2 (*r* = 0.42, *df* = 34, *p* = 0.011). There were no significant differences in overall VISQ scores between T1 and T2.

### Relations to Mood and Temporal Thinking

No significant correlations were observed between mean levels of happiness and either generally endorsed or randomly sampled VISQ scores (all *r* < 0.15, *p* > 0.30). Greater anxiety scores were associated with Other People scores during random sampling (*r* = 0.30, *df* = 49, *p* = 0.032) and, at trend level, T1 endorsements for the same subscale (*r* = 0.27, *df* = 49, *p* = 0.057). Anxiety was also associated with endorsement of Evaluative inner speech at T1 (*r* = 0.29, *df* = 49, *p* = 0.043) but not during random sampling (*r* = 0.05, *df* = 49, *p* = 0.716). Temporal thinking (where higher scores indicated thinking about the future and lower scores indicated thinking about the past) was positively associated with Condensed inner speech (*r* = 0.36, *df* = 49, *p* = 0.01) and negatively associated with Evaluative inner speech (*r* = -0.32, *df* = 49, *p* = 0.023), both at T1. However, these relations were only observed during random sampling for Condensed inner speech (*r* = 0.41, *df* = 49, *p* = 0.003).

## Discussion

The main finding of the present study was that generalized endorsements of inner speech characteristics in many cases did not reliably indicate what is reported via random sampling, contrary to our hypothesis. Generalized reports of inner speech characteristics, gathered by a validated questionnaire (the VISQ), appeared to elicit generally higher levels of endorsement than randomly sampled incident reports. Furthermore, and perhaps most importantly, the correlation between generally endorsed and randomly sampled inner speech depended on the specific kind of inner speech being measured: asking about evaluative and motivational inner speech, compared to other phenomenological characteristics, did not produce consistent self-reports at all between questionnaire and experience sampling methods of measurement.

Participants’ general endorsements of dialog-like inner speech, other people in inner speech, and condensed or fragmentary inner speech showed good test–retest reliability between the start and end of the study, and at levels that were actually higher than in McCarthy-Jones and Fernyhough’s (2011) original study. These three subscales also showed at least some correlation with randomly sampled levels of inner speech collected via experience sampling, but not at the levels of reliability seen for test–retest of the general questionnaire. At the same time, overall endorsement levels for varieties of inner speech were significantly lower during random sampling, supporting Hurlburt et al.’s (2013) argument that asking about inner speech in generalized terms may lead to inflated responses.

Why over-estimation would be occurring is an important question to answer. One thing to note is that the general VISQ does not actually ask about frequency of inner speech: instead, participants rate their level of agreement for what their inner speech is generally like, whenever it occurs. As Hurlburt et al. (2013) note, endorsement of items on this basis provides ambiguous information regarding whether a given experience of inner speech happens often, or happens infrequently but in such a way that makes a participant strongly identify with the scenario described. The random-sampling reports, in contrast, asked about characteristics of inner speech at the moment of the alert, even when inner speech may not have been occurring. Thus, to some degree, reports of inner speech phenomenology are bound to be lower whenever random sampling is used in this way.

However, even with frequency-based responding, over-estimates of inner speech may still be expected from a generalized questionnaire. Self-report questionnaires are notoriously susceptible to various reporting biases, affecting both recall of the phenomenon in question and judgments about how often it occurs (e.g., Houtveen and Oei, 2007; Edmondson et al., 2013). The peak level of a behavior or experience, its level at the end of sampling, and its variability across time can all affect participant accuracy: for instance, participants with more variable chronic pain also tend to overestimate their average pain level compared to those with more consistent pain (Stone et al., 2005).

Consideration of such biases is important for interpreting the results regarding evaluative inner speech. While the other three subscales of the VISQ showed at least some evidence of reliability between the general questionnaire and random sampling, estimates of evaluative and motivational characteristics of inner speech were worryingly divergent. Generalized reports for this factor were not significantly related to random-sampling levels and showed relatively low test–retest reliability for the subsample who completed the VISQ again at the end of the study. This is in distinct contrast with McCarthy-Jones and Fernyhough’s (2011) data, in which general scores for evaluative inner speech showed high test–retest reliability (0.80). Correspondingly, correlations with mood and temporal thinking observed for this characteristic did not hold across generalized and randomly sampled measurements.

Here and in prior studies (McCarthy-Jones and Fernyhough, 2011; Alderson-Day et al., 2014), evaluative inner speech was the VISQ subscale that participants endorsed the most, and was the most likely to be over-estimated. Given prior evidence of variability effects (e.g., Stone et al., 2005), it could be that evaluative inner speech is harder to estimate because of its variance across time compared to other subscales. However, the mean SDs of each subscale do not support this idea (see **Table 1**): Dialogic inner speech was the most variable subscale, rather than Evaluative.

A second reporting bias could arise from the content of evaluative inner speech. Strongly valenced behaviors and states have often been reported to affect accuracy of recall (Shiffman et al., 2008). On both the VISQ (Alderson-Day et al., 2014) and other measures of inner speech (Brinthaupt et al., 2009), the tendency to engage in self-reflective and evaluative processes appears to be linked to negative beliefs and ideas about oneself. If so, evaluative inner speech, compared to other VISQ subscales, may have a greater salience to participants when they think about it in generalized terms, leading to its overestimation on questionnaires. Were this to be the case, discrepancies in inner speech reporting would be expected to be greatest for measures that specifically enquire about positive and negative statements in inner speech (such as the Self-Talk Inventory; Calvete et al., 2005), or in individuals with a tendency toward engaging in more negative, ruminative inner speech behaviors (such as people with depression; Nolen-Hoeksema, 2004).

Exploratory analysis of the relations between inner speech, mood, and temporal thinking indicated two avenues for future study. First, the presence of other people in inner speech—assessed via random sampling—was also associated with greater levels of momentary anxiety. This is consistent with this factor being a general marker for psychopathology, as it has previously been observed to relate to increased trait anxiety, hallucination-proneness, and dissociative tendencies (McCarthy-Jones and Fernyhough, 2011; Alderson-Day et al., 2014). This was also evident for generalized endorsement of other people in inner speech, but only at trend level, suggesting again that random sampling could provide a more accurate assessment of this particular characteristic. Second, higher levels of condensed inner speech (via generalized endorsement and randomly sampled reports) were associated with thinking about the future rather than the past. This is in line with links between inner speech and future thinking observed by D’Argembeau et al. (2011), but suggests that the kind of inner speech being used may differ depending on whether someone is thinking about the past, present, or the future. Condensed inner speech is proposed by Fernyhough (2004) to represent a syntactically and semantically abbreviated form of verbal thinking that results from the internalization of external speech. Its counterpart is expanded inner speech, in which internal talk involves full words and sentences. Speculatively, it is possible that condensed thinking could aid future planning, while more expanded inner speech could be involved in reflecting on the past and specific recall, including reconstructing past events in the greater detail afforded by an expanded linguistic code.

In contrast, no associations were observed between happiness ratings and VISQ scores. This is inconsistent with D’Argembeau et al.’s (2011) finding that inner speech diary reports were more likely to be associated with negative rather than positive thinking, suggesting again that generalized endorsements about inner speech do not show consistent evidence of validity. Happiness has previously been negatively associated with mind-wandering reports collected via experience sampling (Killingsworth and Gilbert, 2010), suggesting that attentional control factors may be more important factors for understanding mood variation than the presence or absence of inner speech.

Some limitations to this study must be acknowledged. First, only one measure of inner speech —the VISQ—was used here, and it is possible that the discrepancy between inner speech reports observed is an artifact of this specific scale rather than inner speech measures more generally. Given the limited validity of other major scales (Uttl et al., 2011), it is not clear that alternative measures would necessarily have performed better. However, the VISQ could definitely be improved: as noted above, the present version of the scale asks participants to answer based on their agreement with general statements rather than specifically indicating frequency. To fully test why incidence estimates of inner speech differ so much between, for example, the studies of Klinger and Cox (1987) and Heavey and Hurlburt (2008) requires an index of inner speech that also asks about the frequency of particular experiences. We are currently in the process of adapting and expanding the VISQ for this purpose, which should remove some of the ambiguity in participants’ responses noted by Hurlburt et al. (2013), and may lead to more accurate generalized data.

Second, for clarity of examining relations between generalized and randomly sampled inner speech, responses to momentary samples were averaged in the present study to provide mean scores. This, however, undoubtedly obscures the level of complexity inherent in experience sampling data. A key question for inner speech research is how it may vary across time in relation to mood and activity factors; further analysis with a larger sample will allow this to be assessed. Third, as is the case for the large majority of recent studies on inner speech, the data collected here are limited to an undergraduate student sample and do not necessarily reflect inner speech characteristics in the general population. The use of research apps like Inner Life should allow researchers to go beyond university-based samples and obtain a more accurate picture of the heterogeneity of inner speech.

Finally, it is important to note that any self-report method, whether gathered by questionnaire or momentary assessment, may be affected by reporting bias. A method such as DES differs from standard self-report techniques and most other experience sampling methods in its attempt to bracket experimenters’ presuppositions and iteratively train participants to avoid their own. Hurlburt et al. (2013) argue that any self-report method is likely to provide inaccurate data on inner experience unless it attempts to do something similar. Notwithstanding the importance of such considerations, we argue here and elsewhere (Alderson-Day and Fernyhough, 2014) that a combination of methods is needed to examine inner speech both on an in-depth, individual level (as in DES) and in larger, representative samples. Examining how data from self-reports may change in their reliability and validity with iterative training is also important for establishing whether people can ‘improve’ in their reporting of inner experience, as DES would hold, although in the present dataset, there were no clear signs of reactivity in response to use of random sampling (cf. Runyan et al., 2013).

In summary, the present article describes the first app-based study of everyday inner speech. Generalized estimates of inner speech can in some cases be reliable indicators of day-to-day characteristics of inner speech, but this varies considerably depending on the kind of self-talk being asked about. It seems likely that memory biases and other confounds affect self-reports about inner speech, particularly for its evaluative and motivational features. This does not mean that self-reports of inner speech are entirely inaccurate, but it strongly suggests that when we ask about inner speech, participants are reporting on the kinds of subjective experiences and processes that are salient and important to them.

## Conflict of Interest Statement

The authors declare that the research was conducted in the absence of any commercial or financial relationships that could be construed as a potential conflict of interest.
